# High-Value Compounds in Papaya By-Products (*Carica papaya* L. var. Formosa and Aliança): Potential Sustainable Use and Exploitation

**DOI:** 10.3390/plants13071009

**Published:** 2024-04-01

**Authors:** Ana F. Vinha, Anabela S. G. Costa, Liliana Espírito Santo, Diana M. Ferreira, Carla Sousa, Edgar Pinto, Agostinho Almeida, Maria Beatriz P. P. Oliveira

**Affiliations:** 1 LAQV/REQUIMTE, Department of Chemical Sciences, Faculty of Pharmacy, University of Porto, R. Jorge de Viterbo Ferreira 228, 4050-313 Porto, Portugal; acvinha@ufp.edu.pt (A.F.V.); acosta@ff.up.pt (A.S.G.C.); lilianaespiritosanto81@gmail.com (L.E.S.); sousasil@ufp.edu.pt (C.S.); aalmeida@ff.up.pt (A.A.); 2 FP-I3ID, Research Institute, Innovation and Development Fernando Pessoa, Faculty of Health Sciences, Fernando Pessoa University, Praça 9 Abril 349, 4249-004 Porto, Portugal; 3 Nutrition and Bromatology Group, Department of Analytical Chemistry and Food Science, Faculty of Science, University of Vigo, E-32004 Ourense, Spain; 4 REQUIMTE/LAQV, ESS, Polytechnic of Porto, Rua Dr. António Bernardino de Almeida, 4200-072 Porto, Portugal; ecp@ess.ipp.pt

**Keywords:** papaya seeds and peel, nutritional composition, fiber contents, mineral profile, vitamin E, phytochemical composition, antioxidant activity

## Abstract

Background: Food waste is a global and growing problem that is gaining traction due to its environmental, ethical, social, and economic repercussions. Between 2022 and 2027, the worldwide papaya market is expected to have a huge increase, meaning a growth in organic waste, including peels and seeds. Thus, this study evaluated the potential use of peels and seeds of two mature papaya fruits as a source of bioactive compounds, converting these by-products into value-added products. Proximate analysis (AOAC methods), mineral content (ICP-MS), free sugars (HPLC-ELSD), fatty acid composition (GC-FID), vitamin E profile (HPLC-DAD-FLD), and antioxidant activity (DPPH and FRAP assays) were evaluated. Results: Both by-products showed high total protein (20–27%), and dietary fiber (32–38%) contents. Papaya peels presented a high ash content (14–16%), indicating a potential application as a mineral source. 14 fatty acids were detected, with α-linolenic acid (30%) as the most abundant in the peels and oleic acid (74%) in the seeds. Both by-products showed high antioxidant activity. Conclusion: Papaya by-products display great potential for industrial recovery and application, such as formulation of new functional food ingredients.

## 1. Introduction

In the last decade, the global production of tropical fruits has been constantly increasing in the main producing areas, mainly in response to the increasing demand [1]. Papaya (*Carica papaya* L.) is a major subtropical and tropical horticulture crop. This fruit is recognized by its nutritional, health and medical characteristics [2,3]. Global papaya production is expected to increase 2.1%/year, reaching 16.6 million tons within five years [4]. The accumulation of papaya’s by-products has increased along with papaya production and consumption. The food industry uses papaya to produce a wide variety of products, for example nectars, juices, jams, pectin, papain, which creates a very significant amount of waste and by-products. In fact, fruit waste can be seen as fruits that are removed from the production line due to the absence of the caliber defined by authorities to be commercialized or due to the presence of superficial damages, whereas food by-products are composed by peels, pomace, stems, leaves, seeds, or pulps generated during industrial chain. Peels plus seeds contribute for around 50% of the papaya mass, whereas seeds constitute approximately 14% [5]. As a result, the management of industrial fruit by-products should have the short-term goal of decreasing food waste, as well as developing procedures that allow its use and consequent appreciation, to obtain economic value [6]. Thus, the use of papaya by-products can contribute to reducing food insecurity, and to improve food sustainability, especially in less developed regions of the world. *Carica papaya* L. shows great therapeutic potential, as well as diversified industrial use [7]. In some Asian countries, bacterial infections, inflammations and stomach pains are treated using the peels, seeds and leaves of *C. papaya* L. [8]. Similarly these parts of the plant are utilized as antioxidant, hepatoprotective [9], and antifungal agents [10]. Those biological activities are related with the presence of bioactive compounds in all parts (edible and inedible) of this fruit. Furthermore, currently, food processing tends to use natural bioactive compounds rather than manufactured ingredients, as the former are effective and safer. Several nutrient and non-nutrient compounds found in papaya leaves, seeds, and peels provide nutritional and health benefits. All papaya by-products are abundant in proteins, oils, fibers, flavonoids, tannins, saponins, alkaloids, anthraquinones, and benzyl isothiocyanate [11,12]. According to Abdel-Hameed et al. [3], papaya seeds possess the capacity to originate oleic oil with good physical and chemical properties, as well as fatty acid profiles that are quite comparable to olive oil. As far as the fruit peels are concerned, it is known that they contain more beneficial chemicals such as β-carotene, lycopene, anthocyanins, and flavonoids, than fruit pulps [13,14]. Some studies have revealed the importance of flavonoids and carotenoids in human health, namely as anti-cancer, and cataract preventive agents, which is related to the antioxidant properties of the compounds and their ability to eliminate free radicals [5,12]. Presently, products derived from plants, which have never been classified as food or medicine, have been used in the form of supplements in the prevention and promotion of health, and in the treatment of specific pathologies, and this trend can be classified as a “bioceutical revolution” [15]. Thus, antioxidant activity from by-products may be used in the manufacture of nutraceuticals and functional foods or ingredients. The large amount of waste generated by papaya processing and its composition allows to believe that food industries would greatly benefit from the recovery of the referred by-products.

The purpose of this research work was to estimate the nutritional and mineral composition, vitamin E and fatty acids profiles, bioactive compounds content, and antioxidant activity of peels and seeds from two commonly consumed papaya varieties (Formosa e Aliança), with the goal of maximizing knowledge of their phytochemical and nutritional value. The health benefits of papaya by-products could lead to the manufacture of nutraceuticals and functional foods.

## 2. Results

### 2.1. Nutritional Composition

Fruit peels and seeds from Formosa and Aliança varieties were analyzed for their nutritional composition (Table 1).

Considerable nutritional differences in both matrices (peels and seeds) and varieties (Formosa and Aliança) were observed.

### 2.2. Mineral Profile

The mineral profile was evaluated by ICP-MS, and results are presented in Table 2.

In general, the content of macro elements, and essential trace elements in the fruit peels exceeded their quantity in the seeds. Still, considerable differences were observed between the two matrices, as well as between fruit varieties.

### 2.3. Vitamin E Profile

Vitamin E has significant applications in the food industry. In this work, new information on vitamin E content is reported in papaya by-products (Table 3).

According to the results reported in Table 3, fruit peels present significantly higher levels of vitamin E. The most prevalent vitamers found in the peels and seeds of both papaya varieties were α-tocopherol and γ-tocopherol.

### 2.4. Fatty Acid Profile

The percentage of fatty acids of the peels and seeds extracted oils from Aliança and Formosa papaya varieties was evaluated by GC-FID, and results presented in Table 4.

In this study, a total of 14 fatty acids were identified, with α-linolenic acid being the most prevalent in the fruit peels and oleic acid in the seeds. Furthermore, other differences between the fatty acid profiles were observed.

### 2.5. Bioactive Compounds and Antioxidant Activity

The total phenolics (TP) and flavonoids (TF) contents and antioxidant activity (DPPH^•^ and FRAP inhibition assays) were analyzed, and results are presented in Table 5.

Regarding the results of the bioactive compounds and antioxidant activity (Table 5), the highest amounts of total phenolics and flavonoids were observed in fruit peels. However, both seeds and peels from different papaya cultivars showed comparable antioxidant activity, despite the difference in the total amount of bioactive compounds between the two matrices.

## 3. Discussion

Papaya fruit by-products contains several bioactive components and offers a high nutritional value that can be further utilized for sustaining food security. Despite their importance, there is still currently few information on papaya seeds and peels as dietary supplements or nutritional ingredients.

Concerning the physical and intrinsic characteristics of Formosa and Aliança varieties, several differences were observed. Physically, Formosa papaya variety is a much larger, weighing an average of 980 g per unit, whereas the smaller Aliança variety weighs ~450 g/unit. However, the variation in the dimensions of the two papaya varieties has no significant effect on the percentage of by-products generated. The by-products (peels and seeds) resulting from the two studied papaya varieties represented approximately 20 to 25% of the fruit’s weight. Aliança papaya seeds constituted higher proportion (~10%) than Formosa variety (~5%) on a fresh matter basis. However, both varieties showed identical percentages of peels, ranging from 20–25% for Aliança and Formosa, respectively. Regarding intrinsic characteristics, both fruit pulps presented identical color and flavor.

Regarding nutritional composition of Formosa and Aliança varieties by-products, the observed differences could be attributed not only to dissimilarity of each matrix and variety, but also to an assortment of factors, including each fruit maturity index at the time of the research. The observed variances may be based on the mentioned parameters, however our samples lacked sufficient information about the state of maturity at the harvesting time, even though they were cultivated in the same territorial region. Moreover, it was expected that variations in nutritional content would be observed between fruit varieties, considering that the macro and micronutrients contents are directly related to each plant’s primary metabolism.

Regarding the results, for ashes the values were significantly higher in fruit peels (13.8–15.8%) when compared to the seeds (8.6–9.5%). These results are higher than those described by other authors in a similar investigation [16]. Even accounting for the variations among papaya varieties, Santos et al. [17] additionally noted that the peels of Calimosa and Havai cultivars (11.31% and 11.85%) had greater ash content than seeds (6.94% and 7.73%), yet lower than the referred in this study. Another observed fact focuses on the converse correlation between moisture and ash contents, in agreement with some authors, who confirmed the same inversely proportionate relationship [18]. Despite the ash content of the papaya seeds was lower than peels, our values were higher than those described in other fruit seeds, including gourd seeds (6.3%), pumpkin seeds (3.8%), watermelon seeds (5.3%), and musk melon seeds (4.5%) [19], indicating the potential application of papaya by-products as mineral sources.

Protein is a necessary component of the diet for both animal and human survival, and its primary function is to supply the appropriate amount. Regarding crude protein content, Table 1 shows that Aliança fruit variety exhibited the maximum crude protein content in peels (26.6%) and seeds (25.6%), whereas the minimum was observed in the Formosa peels (19.9%). Similar protein contents were described by Moses and Olanrewaju [20], in papaya seeds and peels (13.6% and 27.4%, respectively) collected in Nigeria. Nevertheless, lower values were described by other authors, with 14.6% and 23.6% in papaya peels and seeds, respectively, from Bangladesh [21]. Although the amounts of crude protein remain high, even in accordance with other studies, it must be considered the analytical process used to determine the total crude protein content. Indeed, this content comprises both protein and non-protein components. As already mentioned, non-protein nitrogenous compounds such as alkaloids and isothiocyanates are described in papaya by-products [11,12], that may overvalue the protein contents. Another element that could influence the total crude protein contents is the availability of several conversion factors that researchers may employ. In truth, there is no agreement in the application of a precise conversion factor for fruit peels and seeds, and there may be divergences in the results obtained in different studies. Moreover, following this study, as analysis of the amino acid profile is proposed due to the significant protein level detected. Regarding fat contents, a higher amount for both fruit seed varieties was observed (~25%), with no significant differences. In fact, as expected, the fat content is mainly concentrated in the seeds. The results of this study, although slightly inferior, agree with Abdel-Hameed et al. [3] and Kumoro et al. [5], both reporting fat content in papaya´s seeds ranging from 28% and 32%, respectively. In other papaya varieties (Calimosa and Havai) levels of 28.1% and 28.6% for seeds, and 2.1% and 2.4% for the peels were described [17]. There are also some studies reporting lower fat contents in the same matrices [16,22]. Despite the differences found between the different authors, the high fat content observed in the studied papaya varieties seeds indicate them as a good source of oil, which can serve as energy source.

The total carbohydrate content was also determined, and Formosa variety presented higher total carbohydrates (62.8% in peels and 41.6% in seeds) than Aliança (54.8% and 40.5% for peels and seeds, respectively). Higher total carbohydrate contents were reported in ripe papaya by-products (64.2% in peels, and 55.4% is seeds) [21], indicating that the ripening fruits index increases their overall carbohydrates content. Regarding dietary fiber, as shown in Table 1, both fruit by-products presented high levels, ranging from 31.7% to 37.8%. However, Formosa seeds showed the highest values of insoluble and soluble fiber (31.4% and 6.4%, respectively). These results are inconsistent with other authors, mainly in fruit peels, which confirmed lower dietary fibers in ripe papaya peels (9.1%) and seeds (5.3%) [21], however in agreement with Santos et al. [17] who reported values between 7.8% and 8.8% in Calimosa and Havai papaya seeds and 33.5% to 34.7% in fruit peels. Also, higher total fiber content was described in papaya peels (56.1%) collected in Thailand [23]. Despite the differences that have been reported, dietary fiber consumption is important due to benefits in human health and proper body function. Also, consuming the recommended amount of dietary fiber helps lower the risk of common health problems such as constipation, obesity, and diabetes [24]. Greater notability has been given to natural food sources due to their high contents in dietary fiber and phytochemical compounds, as a healthier food choice for glycemic control [23]. Likewise, the quantification of free sugars was carried out. Of the studied sugars (glucose, fructose, sucrose, maltose and rhamnose), only two, fructose, and glucose, were detected in both fruit by-products, with significantly higher levels in the fruit peel. Fructose content in papaya peels ranged from 12.0% to 18.4%, for Aliança and Formosa varieties, respectively. Significantly lower values were observed in the seeds of these two varieties (4.3% and 3.3%). Glucose contents were also higher in peels, ranging from 10.1% to 16.2%, predominant in the Formosa variety, and significantly lower values were found in the seeds, with a slight variation from 3.9% (Formosa) to 4.6% (Aliança). It is known that free sugars play an important role in fruit quality, and during their ripening, there is a predisposition to an increase in total soluble solids and soluble sugar levels in the pulp and peels of the fruits. Moreover, several studies have documented a direct relationship between the free sugar content with fruit sweetness and maturation index, as a crucial aspect of fruit quality [25,26]. In addition to the nutritional contribution, the assessment of free sugar levels is important, as they can regulate a series of functions, including fruit hormonal balance, photosynthesis, nitrogen intake, defense mechanisms and secondary metabolism [26]. Thus, in our investigation, papaya by-products exhibited nutraceutical values, highlighting their importance in diets for humans. According to the results, the composition of papaya by-products varies greatly between studies, which can be attributed to differences in varietals or cultivars, ripening phases, and edaphoclimatic conditions [16]. Recent studies have already highlighted the importance of papaya seeds as a functional ingredient. Lira et al. [27] described the importance of papaya seed flour as a functional ingredient in the production of cupcakes, reporting a considerable increase in fiber content, as well as protein and fat contents. Abdel-Hameed et al. [3], in an identical study, confirmed that cakes enhanced with papaya seeds exhibit immunostimulant effects and protect against CCl4-induced immunotoxicity and hepatotoxicity in rats. Also, Joymak et al. [23], affirmed that papaya peels can be considered as a good source of nutrients for the development of healthy products. Indeed, papaya peel varieties studied are rich in ash, protein, and insoluble fiber. Thus, the application of papaya peels in food industry, for instance, in flour production is emphasized as a potential added-value ingredient.

No recent data on the mineral profile of papaya by-products were found in literature. Minerals are, in part, responsible for the proper functioning of the immune system [28]. Moreover, mineral intake is vital for the optimal function of the innate immune system as well as adaptive immune defense components; this includes defense mechanisms against infections as well as the long-term balance of pro- and anti-inflammatory regulation [28]. Regarding the content of macroelements, an identical profile between the two by-products studied was found; potassium (K) has the highest value, ranging from 41.2–60.4 mg/g, followed by calcium (Ca) with a variation from 7.93–12.7 mg/g and magnesium (Mg) ranging from 2.68–4.40 mg/g. Comparing these results with the reported ones from Chukwuka et al. [16] and Morais et al. [29], papaya peels presents higher K and Na contents, whereas the seeds have higher Mg content. The same authors stated that K and Ca were the most abundant macro elements in both peels and seeds papaya varieties, which are in accordance with the presented results. As regards elemental trace minerals, iron (Fe) and zinc (Zn) were predominant in both by-products. The selenium (Se) content was higher in papaya peels (doubling its content), highlighting its possible application as a natural source of vital minerals. Selenium plays a substantial role in the human organism, namely in its binding to proteins (selenoproteins), thus supporting antioxidant defense systems. Addicionally, the reported findings are similar to those presented by Martial-Didier et al. [30], who reported potassium as the most abundant mineral presented in the peels of Solo papaya variety. Minerals, which are abundant in papaya by-products, are necessary for teeth structure (Ca) [31], and bones (Ca, Mg, Mn, B) [32], while microminerals like Cu, Fe, Mn, Se, and Zn are crucial for enzyme structure and biological functions [33].

In recent years, functional foods, plant-derived nutraceuticals, and dietary supplements have been including health-promoting components. They all include bioactive substances with positive health effects in addition to nutritious components. High dietary intakes of vitamin E have been associated with a decreased risk of several diseases, beyond its antioxidant effect [34]. Considering these advantages, this work has included the determination of vitamin E content in papaya by-products. Few studies addressed its quantification, only reporting the vitamin E content of papaya as undetectable or in low concentrations (~0.3 mg/100 g) [34]. According to the obtained results, papaya peels contain considerable vitamin E contents (65.0 to 100.6 mg/100 g). Significantly lower levels were found in the seeds (3.5–4.1 mg/100 g) with α-tocopherol being the vitamer present in greater quantities. Furthermore, only α-tocopherol and γ-tocopherol were detected in the seeds, in similar amount like those reported by Maisarah et al. (4.1 mg/100 g) [22]. Currently, the effects of natural antioxidants are studied to enhance the benefits of the processed food products in which they are incorporated. Tocopherols (vitamin E) are commonly used during food processing, notably to avoid the oxidation of fats and oils, as well as the discoloration and degradation of oxygen-sensitive nutrients [35]. Following this context, Najjar et al. [36] demonstrated that papaya seeds flour can improve quality in biscuit manufacture due to vitamin E contents. Thus, the presented results suggest the use of these by-products with potential application in enriched flours, presenting several possible applications in food industry.

The lack of information about fatty acids in *Carica papaya* L. species, combined with the possibility to obtain new food sources of fatty acids, aroused the interest in analyzing the lipid composition of papaya by-products. The percentage of different fatty acids varied across fruit cultivars, and among different by-products of the same cultivar. According to the results presented in Table 4, a total of 14 fatty acids were detected, with α-linolenic acid (30.3%) being the most abundant in the fruit peels and oleic acid (73.6%) in the seeds. Regarding fruit peels, the dominant fatty acid was α-linolenic, ranging from 28–30%; followed by palmitic acid (25–27%); linoleic acid (9–14%); and oleic acid (9–10%). Heptanoic acid was observed only in papaya fruit peels in traces, while cis-11-eicosanoic was only found in seeds of both cultivars. Tricosanoic and lignoceric acids, both saturated fatty acids (SFA) were quantified in papaya peels. Regarding the seeds fatty acids, oleic acid was the predominant one (73–74%) with significant differences between fruit varieties (*p* < 0.05). Interestingly, peel samples possess the highest SFAs contents (42 and 45% for Aliança and Formosa, respectively), and PUFAs contents (39–43% for Formosa and Aliança, respectively). Fruit seeds have the highest content of MUFAs (73–74% for Aliança and Formosa, respectively). Thus, papaya seeds can be consumed directly or after industrial processing and could be an alternative for other natural products with comparable or lower levels of oleic acid, such as sunflower (80%), canola (75%), safflower (77%), and olive (70–83%) [3]. Also, papaya seeds contain lower amounts of linoleic acid, allowing a reduction in oxidation potential, and consequently, a longer shelf life to several products, which is an advantage for potential industrial applications in food, pharmaceutical, and cosmetic industries. Mesquita et al. [2], Yao and Xu [37], and Santana et al. [38] reported similar results for papaya oil seeds obtained from several papaya varieties. According to these researchers, papaya seeds may contain 30–40% of oil, making them as economically useful by-product. Recognizing the fatty acid composition in both functional and nutritional properties of oils, oleic acid was reported as the main MUFA in apple (43 g/100 g) and pear (57 g/100 g) seed oils [39], significantly lower than those reported in this work.

Polyphenols have gained popularity in recent decades, owing mostly to their potential benefits to human health, resulting from their significant bioactivities (antioxidant, anti-inflammatory, antimicrobial, antidiabetic, among others) [2,11,40]. These bioactive molecules can be collected from by-products using several technologies and used to generate a variety of valorized products, such as functional foods or dietary supplements. Papaya fruits are commonly recognized to contain a substantial number of phenolic compounds, including phenolic acids and flavonoids, which possess strong antioxidant activity [41]. It is expected always higher concentrations of total phenolics than total flavonoids contents, and the obtained results highlight this fact, since in all by-products extracts a higher content of total phenolics was observed. Thus, our findings were not consistent with others authors. For instance, Gaye et al. [42] described higher total phenolic contents in papaya peels ranging between 15.5 and 23.6 mg GAE/g, depending on fruit variety. In the presented research, the content of total phenolics in papaya peels ranged from 8.0 to 8.9 mg GAE/g, significantly higher to those described by Zhou et al. [41]. The content of bioactive compounds in both by-products from the same variety can be compared. Aliança cultivar presented higher phenolic contents in peels (8.9 mg GAE/g) and seeds (4.7 mg GAE/g) than Formosa (8.0 and 4.3 mg GAE/g for peels and seeds, respectively). Also, the flavonoids content followed the same order, presenting higher amounts in fruit peels (2.3 mg CE/g and 2.5 mg CE/g for Formosa and Aliança varieties, respectively). No differences were observed in fruit seeds, ranging from 1.1 to 1.3 mg CE/g. Previously, Martial-Didier et al. [30] reported a total flavonoids content in the peels of Solo papaya variety of 5.6 mg QE/g, which cannot be compared to the presented results. Despite the differences in bioactive compound contents, both papaya by-products exhibit potential as nutraceutical ingredients. As a matter of fact, according to Subandi et al. [43], papaya seeds contain two different kinds of flavonoids that may inhibit pancreatic lipase. Zhou et al. [41] claim that papaya peels possess a notably larger quantity of bioactive compounds than the seeds.

To determine the antioxidant activity of food samples, several techniques are frequently used. The antioxidant potential of the peels and seeds of papaya was determined in the current study using DPPH and FRAP assays. Table 5 showed that Aliança fruit seeds presented the highest DPPH value (30.3%), followed by Formosa fruit seeds, with 28.5%, which was higher than Aliança peels (26.8%), and Formosa peels (26.2%). Results showed that the DPPH value of papaya by-products was in the order of seed > peel. However, according to Kadiri et al. [44], the papaya peel exhibited higher free radical scavenging activity, followed by the seed, which is the opposite of the observed in this study. The FRAP assay was also used to assess the antioxidant activity in papaya by-products, using a ferric tripyridyltriazine (Fe^III^-TPTZ) complex. In the present study, the highest FRAP capacity was observed in Aliança variety for the peel and seed portions (89.4 μmol FSE/g, and 79.2 μmol FSE/g, respectively). In the peels and seeds, the FRAP values shared the same pattern (peels > seeds). However, in the peel comparison, the Aliança variety presented higher antioxidant activity, followed by Formosa variety. Despite everything, papaya by-products showed high antioxidant activity, which is directly linked to the existence of compounds with bioactive properties.

## 4. Materials and Methods

### 4.1. Chemicals

The reagents used were of analytical grade and were as follows: anhydrous sodium sulfate (Na_2_SO_4_) from Merck (Darmstadt, Germany); petroleum ether from Sigma Chemical Co. (St. Louis, MO, USA); sand from VWR International, (Leuven, Belgium). Kjeldahl catalyst pellets (Na_2_S_2_O_8_/CuSO_4_) and 98% concentrated sulfuric acid (H_2_SO_4_) from Merck (Darmstadt, Germany); sodium hydroxide (NaOH) from VWR International (Leuven, Belgium); boric acid (H_3_BO_3_) from Panreac (Barcelona, Spain); and sulfuric acid (H_2_SO_4_) with a concentration of 0.5 M from Carlo Erba (Val de Reuil, France). Acetonitrile HPLC from Riedel-de Haën (Honeywell, Charlotte, NC, USA); fructose, glucose, sucrose, maltose, and rhamnose from Sigma Chemical Co. (St. Louis, MO, USA). Enzyme Kit (α-amylase, protease and amyloglucosidase) from Sigma Chemical Co. (St. Louis, MO, USA); anhydrous disodium hydrogen phosphate and sodium dihydrogen phosphate from Merck (Darmstadt, Germany); sodium hydroxide and 1.0 M hydrochloric acid from Carlo Erba (Val de Reuil, France); ethanol from Aga (Prior Velho, Portugal); acetone from VWR International (Leuven, Belgium). High purity nitric acid (HNO_3_, ≥69% *w*/*w*) and hydrogen peroxide (H_2_O_2_, ≥30% *v*/*v*) for trace metal grade were purchased from Fisher Scientific, (Leicestershire, UK). The certified reference materials (CRM) White cabbage (BCR-679) and Hay Powder (BCR-129) were purchased from European Commission, Joint Research Centre (Brussels, Belgium). Periodic table mix 1 for ICP, periodic table mix 2 for ICP, periodic table mix 3 for ICP, were purchased from Sigma-Aldrich (Buchs, Switzerland). Matrix reference material (MRM) Enviromat Drinking water-high level of concentration was purchased from SCP SCIENCE (Quebec, QC, Canada). Calcium, iron, magnesium, and sodium standards for FAAS were purchased from Fluka (St. Louis, MO, USA), while potassium standard was purchased from Sigma-Aldrich. n-Hexane HPLC from Merck (Darmstadt, Germany); 1,4-dioxane from Sigma Chemical Co. (St. Louis, MO, USA); tocopherols and tocotrienols standards: α, β, γ, δ-tocopherol and α, β, γ, δ-tocotrienol from Calbiochem (La Jolla, CA, USA); internal tocol standard: 2-methyl-2-(4,8,12-trimethyl tridecyl)-chroman-6-ol from Matreya Inc. (PA, USA). Dichloromethane from VWR International (Leuven, Belgium); potassium hydroxide from Panreac (Barcelona, Spain); n-hexane (HPLC) and anhydrous sodium sulfate from Merck (Darmstadt, Germany); boron trifluoride (BF3, 14% in methanol) from Sigma Chemical Co. (St. Louis, MO, USA); mixture of fatty acid standards (FAME 37, Supelco, Bellefonte, PA, USA). Absolute Ethanol from Fisher Chemical (Loughborough, England, UK); sodium carbonate (Na_2_CO_3_), gallic acid, catechin, DPPH^•^ (1,1-diphenyl-2-picrylhydrazyl radical), trolox, TPTZ (2,4,6-tripyryl-s-triazine), sodium nitrite (NaNO_2_), aluminum chloride (AlCl_3_), ferrous sulfate (FeSO_4_), sodium acetate, glacial acetic acid and ferric chloride from Sigma Chemical Co. (St. Louis, MO, USA); Folin-Ciocalteau reagent from Merck (Darmstadt, Germany); 1.0 M sodium hydroxide (NaOH) Carlo Erba (Val de Reuil, France).

### 4.2. Material and Sample Preparation

Approximately about 10 kg of fresh fruits of papaya (*Carica papaya* L.) varieties, Formosa and Aliança cultivated in Quinta de Santa Terezinha, Paraíba, Brazil (7°5′5″ S, 37°26′30″ W (−7.084722, −37.441667) were purchased in Porto (Portugal), in 2020, in two different supermarket chains. Fruit seeds and peels were collected manually, stored separately in sample bottles at −80 °C, and subsequently lyophilized (Telstar Cryodos-80 Terrassa, Barcelona, Spain). The by-products were ground in a mill (Grindomix GM200, Rech, Germany) to obtain homogeneous powder samples. After, dried powdered samples were used to carry out proximate and phytochemical analyzes. All determinations were carried out in triplicate.

### 4.3. Nutritional Analysis

Moisture was quantified with an infrared balance (Scaltec model SMO01, Scaltec Instruments, Heiligenstadt, Germany). The other nutritional assays were achieved in agreement with AOAC procedures [45]. Summarily, ashes were determined after incineration (500 °C). Total lipids and crude protein were estimated by the Soxhlet and the Kjeldahl methods, respectively. Total and insoluble fiber content was evaluated through the enzymatic-gravimetric method [45]. The soluble fiber and total carbohydrates were determined by difference. The results were expressed as g/100 g of dry weight (dw).

### 4.4. Determination of Free Sugars by HPLC

Sample (500 mg seeds; 250 mg peels) was placed in a Falcon tube, and the volume of 10 mL was attained with deionized water. The tubes content was homogenized for 30 min, followed by centrifugation (Megafuge 16, Heraeus, Hanau, Germany), 5000 rpm, 15 min. Samples were filtered into injection vials using syringe filters. The free sugar content of each sample was determined by an HPLC system (Jasco, Tokyo, Japan) equipped with an evaporative light scattering detector (ELSD), following a previously validated protocol [46]. Sugars were identified based on the retention time of known standards and quantified based on the calibration curves obtained by plotting peak area versus the concentration of each sugar. The concentrations of the standards ranged from 0.2 to 6.0 mg/mL, for glucose (R^2^ = 0.9996), fructose (R^2^ = 0.9991), sucrose (R^2^ = 0.9994), maltose (R^2^ = 0.9992), and rhamnose (R^2^ = 0.9995). The separation was carried out on a Shodex Asahipak NH2P-50 4E column (4.6 mm I.D. × 250 mm), using an isocratic system with two eluents: (A) water (25%) and (B) acetonitrile (75%). Samples were eluted at a flow rate of 0.5 mL/min for 20 min at 30 °C. Each determination was repeated in triplicate and the results were presented in g/100 g dw.

### 4.5. Macro and Trace Elements Composition

Mineral analysis was achieved in agreement with Pinto et al. procedure [47]. In short, 250 mg of each sample was digested in an MLS-1200 Mega high-performance microwave digestion unit equipped with an HPR-1000/10 S rotor (Milestone, Sorisole, Italy), with nitric acid (65%) and hydrogen peroxide (30%) solutions. After digestion, the mixture was diluted to 25 mL with ultrapure water. A Perkin Elmer (Überlingen, Germany) 3100 flame (air-acetylene) atomic absorption spectrometer was used to determine the macro and trace elements contents. The calibration standards were obtained from the dilution of standard stock solutions of each element with a concentration of 1000 mg/L of Ca, Na, Mg, Fe or K. For elemental analysis, an iCAP™ Q ICP-MS—inductively coupled plasma mass spectrometer (Thermo Fisher Scientific, Bremen, Germany) (ICP-MS) was used. Calibration standards in the range of 0.5–200 μg/L were obtained from the commercial 10 mg/L PlasmaCAL SCP-33-MS multi-element standard solution. Internal Standards (IS) solution was prepared at a concentration of 100 μg/L by appropriate dilution of AccuTrace™ ICP-MS-200.8-IS-1 solution (100 mg/L of Sc, Y, In, Tb and Bi). The next elemental isotopes were monitored: ^7^Li, ^9^Be, ^11^B, ^27^Al, ^48^Ti, ^51^V, ^52^Cr, ^55^Mn, ^59^Co, ^60^Ni, ^65^Cu, ^66^Zn, ^75^As, ^82^Se, 85Rb, ^88^Sr, ^90^Zr, ^98^Mo, ^111^Cd, ^118^Sn, ^121^S, ^133^Cs, ^137^Ba, ^182^W, ^208^Pb, and ^209^Bi. All determinations were executed in triplicate and the results related to dry weight (dw).

### 4.6. Lipid Fraction Extraction

The lipid fraction of each sample was extracted in agreement with the procedure of Alves et al. [48], with minor modifications. About 150 mg of sample were mixed with 75 μL of BHT 0.1% (*m*/*v*), 50 μL of tocol (internal standard, 0.1 mg/mL) and 1 mL of absolute ethanol. The obtained solution was mechanically homogenized for 30 min in an orbital vortex mixer (VV3, VWR International, Darmstadt, Germany). Subsequently, the homogenized solution was combined with 2 mL of n-hexane (HPLC grade), and homogenized for an additional 30 min. At this time, 1 mL of NaCl 1% (*m*/*v*) was mixed. After centrifugation (5000 rpm for 5 min) (Heraeus Labofuge A, Hanau, Germany), the supernatant was collected and stored. The residue was re-extracted with 2 mL of n-hexane for 30 min, and centrifuged (5000 rpm for 5 min). The supernatant was collected and added to the previous extract. The resulting solution was mixed with enough anhydrous sodium sulphate (Na_2_SO_4_), and the obtained mixture was then centrifuged (5000 rpm for 5 min). The supernatant was collected, evaporated in nitrogen stream until 1 mL and injected in a HPLC-DAD-FLD (high-performance liquid chromatography coupled to diode array detector and fluorescence detector) system for determination of the vitamin E profile.

#### 4.6.1. Vitamin E Profile by HPLC-DAD-FLD

The vitamin E profile of the lipid fractions was estimated by HPLC-DAD-FLD, in agreement with the procedure of Alves et al. [48]. The chromatographic analysis was performed in a HPLC system (Jasco, Tokyo, Japan) equipped with a MD-2015 multiwavelength diode array detector (DAD) coupled to a FP-2020 fluorescence detector (Jasco, Tokyo, Japan), programmed for excitation at 290 nm and emission at 330 nm. The chromatographic separation of the compounds was obtained using a normal phase SupelcosilTM LC-SI column (75 mm × 3.0 mm, 3.0 μm) (Supelco, Bellefonte, PA, USA). The eluent utilized was 1.2% 1,4-dioxane in n-hexane (HPLC grade), at a flow rate of 0.600 mL/min. The identification of vitamin E vitamers was achieved by comparison of their retention times with those of standards (α, β, γ, δ-tocopherols and α, β, γ, δ-tocotrienols), based on their UV spectra. Quantitative determination was achieved based on the fluorescence signals, converted to concentration units through calibration curves plotted from commercial standards of each compound, resorting to the internal standard method. The results were expressed as mg/100 g of sample (dry weight).

#### 4.6.2. Fatty Acids Composition Analysis by GC-FID

The fatty acids (FA) profile was performed with a gas chromatograph coupled with flame ionization detector (GC-FID) after derivatization of the lipid fraction extracts to FA methyl esters according to ISO 12966-2017 [49], using KOH in methanol (0.5 M) and BF_3_ in methanol (14%). For separation, the equipment utilized was a GC-2010 Plus gas chromatograph (Shimadzu, Tokyo, Japan), in agreement with the procedure of Nunes et al. [50], with minor changes. The work equipment was an automatic sampler and a split/splitless auto injector (AOC-20i Shimadzu) operating with a 50:1 split ratio at 250 °C (injection), a CP-Sil 88 silica capillary column 50.0 m × 0.25 mm inner diameter and 0.20 μm film thickness from Varian (Middelburg, The Netherlands) and a Flame Ionization Detector (Shimadzu, Tokyo, Japan) at 270 °C. The injection volume was 1.0 μL, the carrier gas used was helium (3.0 mL/min) and the analyses were achieved adopting the next programmed temperature: 120 °C held for 5 min, 2 °C/min to 160 °C held for 15 min and 2 °C/min to 220 °C held for 10 min. The FA methyl esters were identified by comparison with a standard mixture (FAME 37, Supelco, Bellefonte, PA, USA) and data were analyzed based on relative peak areas. Results of FA were expressed in relative percentage of total FA.

### 4.7. Bioactive Contents and Antioxidant Activity

#### 4.7.1. Extracts Preparation

For the extraction preparation, ~250 mg of fruit peels and ~500 mg of fruit seeds were combined with 50 mL of an hydroalcoholic solution (50:50 *v*/*v*). The mixture was kept stirring at 40 °C for one hour. After, the mixture was filtered through Whatman No. 4 filter paper. 1 mL of each filtrate solution was transferred to 10 mL tubes, and stored at −20 °C, for subsequent analysis of bioactive compounds and antioxidant activity. All measurements were achieved in triplicate.

#### 4.7.2. Total Phenolics and Total Flavonoids Contents

The Folin-Ciocalteu reagent was used to measure the total phenolics content (TPC) following analytical methodology, with slight changes [51]. Briefly, 150 μL of Folin-Ciocalteu reagent (1:10) and 120 μL of Na_2_CO_3_ aqueous solution (7.5% *m*/*v*) were mixed with 30 μL of each sample extract. The mixture was then incubated at 45 °C, for 15 min, followed by 30 min of incubation in the dark, at room temperature, before absorbance readings at 765 nm using a Synergy HT Microplate Reader (BioTek Instruments, Inc., Winooski, VT, USA). TPC was quantified from a calibration curve prepared with gallic acid as a standard (5–100 mg/L; R^2^ = 0.9992), and results were expressed as mg of gallic acid equivalents (GAE)/g of dry weight.

Total flavonoids content (TFC) was calculated using a colorimetric method according to the analytical procedure described by Costa et al. [49]. To begin, a solution was prepared by combining 1 mL of each sample extract with 300 μL of 5% sodium nitrite (NaNO_2_) in 4 mL of distilled water. Following a 5-min interval at room temperature, 300 μL of 10% aluminium chloride (AlCl_3_) were added, and after 1 min, 2 mL of sodium hydroxide (NaOH 1 M) and 2.4 mL of distilled water were incorporated in the mixture. Catechin was utilized to plot a standard curve (2.5–400 mg/L; R^2^ = 0.999). The absorbance at 510 nm was measured using a Synergy HT Microplate Reader (BioTek Instruments, Inc., Winooski, VT, USA). Results were expressed as mg of catechin equivalents (CE)/g of dry weight.

#### 4.7.3. Antioxidant Activity

The samples’ ability to scavenge DPPH was assessed using the DPPH^•^ scavenging radical method, according to an established procedure [51]. The experiment was initiated by combining 30 μL of Trolox standard (562 mg/L)/blank/diluted extract (1:10) with 270 μL of the DPPH^•^ solution (6.1 × 10^−5^ M) earlier prepared. The decrease of DPPH^•^ was monitored with a microplate reader Synergy HT (Biotek Instruments, Inc., Winooski, VT, USA) in time intervals of 10 min and absorption at 525 nm, to study the kinetic reaction. The reaction endpoint was achieved in 20 min. The following method was used to find the inhibitory percentage for each sample extract.

The used ferric-reducing antioxidant power (FRAP) assay was previously described [50]. In summary, 265 μL of the FRAP reagent (containing 0.3 M acetate buffer, 10 mM TPTZ solution, and 20 mM ferric chloride) was mixed with 35 μL of ferrous sulphate standard (5–600 μM)/blank/diluted sample extract (1:10). The final mixture was stored for 30 min at 37 °C in the dark, after which the absorbance was measured using a Synergy HT Microplate Reader (BioTek Instruments, Inc., Winooski, VT, USA), at 595 nm. A calibration curve was plotted with ferrous sulphate (5–600 μM; R^2^ = 0.999) and ferric reducing antioxidant power was expressed as μmol of ferrous sulphate equivalents (FSE)/g of dry weight.

### 4.8. Statistical Analysis

The experimental data was analyzed in triplicate, and the results were presented as the mean ± standard deviation. Statistical analysis of the data was achieved with IBM SPSS Statistic (version 26 for Windows, IBM Corp. Armonk, NY, USA). One-way ANOVA was used to assess significant differences observed between samples, followed by Turkey´s HSD to make paired comparisons between means with a significance level of 5% (*p* ≤ 0.05).

## 5. Conclusions

The growing interest of the agrifood industry in recycling has led to a rise in the number of research studies on sources of natural compounds that can be added as functional ingredients. These are crucial in enhancing the complete exploitation of sources of industrial residues produced in high amounts. The obtained results highlighted the nutritional and chemical profile of papaya by-products (peels and seeds), emphasizing the high contents of crude protein, ashes, dietary fiber, mineral profile and MUFAS and PUFAS contents. Thus, using the remaining peels and seeds would be beneficial to develop new enriched foods, growing understanding of their industrial integration on the food industry. In addition, future studies will be carried out testing the amino acid profile, as well as the safety of extracts, by carrying out in vitro tests on cytotoxicity and genotoxicity assays, anti-proliferative and antioxidant properties in cell-based assays, to further improve the bioactive quality of these fruit by-products.

## Figures and Tables

**Table 1:** Nutritional profile of the peels and seeds of two papaya varieties (Aliança and Formosa). Results are expressed in g/100 g dry weight (dw).

Nutrient	AP	FP	AS	FS
Moisture	5.31 ± 0.35 ^c^	6.65 ± 0.22 ^b^	8.18 ± 0.32 ^a^	8.14 ± 0.40 ^a^
Ash	15.82 ± 0.02 ^a^	13.83 ± 0.06 ^b^	8.62 ± 0.02 ^d^	9.50 ± 0.08 ^c^
Crude Protein	26.57 ± 0.03 ^a^	19.86 ± 0.13 ^d^	25.58 ± 0.21 ^b^	23.67 ± 0.31 ^c^
Total Fat	2.85 ± 0.30 ^b^	3.47 ± 0.39 ^b^	25.30 ± 0.23 ^a^	25.25 ± 0.34 ^a^
Total Carbohydrates	54.75 ± 0.33 ^b^	62.84 ± 0.32 ^a^	40.51 ± 0.02 ^c^	41.57 ± 0.62 ^c^
Total Dietary Fiber	34.76 ± 0.05 ^b^	31.69 ± 0.05 ^d^	33.78 ± 0.05 ^c^	37.78 ± 0.08 ^a^
Insoluble Dietary Fiber	31.71 ± 0.03 ^a^	30.22 ± 0.04 ^b^	29.81 ± 0.06 ^b^	31.40 ± 0.15 ^a^
Soluble Dietary Fiber	3.05 ± 0.06 ^c^	1.47 ± 0.06 ^d^	3.97 ± 0.08 ^b^	6.38 ± 0.17 ^a^
Free Sugars				
Glucose	10.10 ± 0.16 ^b^	16.17 ± 0.38 ^a^	4.58 ± 0.10 ^c^	3.85 ± 0.23 ^c^
Frutose	11.95 ± 0.25 ^b^	18.39 ± 0.47 ^a^	4.25 ± 0.11 ^c^	3.32 ± 0.26 ^c^

Results expressed in g/100 g dry weight. Values are presented as mean ± standard deviation *(n* = 3). Values that do not share the same letter are significantly different (*p* < 0.05), determined by ANOVA followed by the Tukey HSD test. AP—Aliança peels, FP—Formosa peels, AS—Aliança seeds; FS—Formosa seeds.

**Table 2:** Total content of essential, non-essential and toxic trace elements in papaya by-products.

	AP	FP	AS	FS
Essential trace elements				
Fe (µg/g)	67.73 ± 0.66 ^b^	57.7 ± 2.3 ^b^	79.6 ± 4.5 ^a^	60.2 ± 3.8 ^b^
Cu (µg/g)	4.440 ± 0.067 ^c^	5.095 ± 0.085 ^b^	6.50 ± 0.14 ^a^	6.64 ± 0.18 ^a^
Zn (µg/g)	28.56 ± 0.53 ^a^	22.20 ± 0.31 ^c^	29.63 ± 0.87 ^a^	24.80 ± 0.58 ^b^
Mn (µg/g)	72.0 ± 1.3 ^a^	31.96 ± 0.16 ^c^	45.5 ± 1.1 ^b^	33.17 ± 0.69 ^c^
Mo (µg/g)	11.17 ± 0.19 ^a^	6.0 ± 1.7 ^b^	3.28 ± 0.69 ^c^	3.17 ± 0.25 ^c^
Co (ng/g)	56.7 ± 2.1 ^c^	286.8 ± 3.4 ^a^	26.8 ± 3.1 ^d^	152.6 ± 5.6 ^b^
Cr (ng/g)	203.5 ± 1.7 ^a^	135.43 ± 0.62 ^a^	128.1 ± 2.3 ^a^	124 ± 20 ^a^
Se (µg/g)	0.302 ± 0.010 ^a^	0.228 ± 0.016 ^b^	0.177 ± 0.011 ^b,c^	0.182 ± 0.023 ^c^
Non-essential and toxic trace elements				
Al (µg/g)	22.8 ± 6.0 ^b^	38.8 ± 2.3 ^a^	1.95 ± 0.48 ^c^	<LoD
As (ng/g)	30.2 ± 8.3 ^a^	29.3 ± 5.0 ^a^	28.7 ± 7.4 ^a^	<LoD
B (µg/g)	27.75 ± 0.67 ^a^	27.62 ± 0.55 ^a^	11.51 ± 0.42 ^b^	11.46 ± 0.40 ^b^
Ba (ng/g)	1456 ± 17 ^a^	954.0 ± 8.2^c^	1274 ± 60 ^b^	1506 ± 25 ^a^
Be (ng/g)	<LoD	<LoD	<LoD	<LoD
Bi (ng/g)	n.d.	n.d.	n.d.	n.d.
Cd (ng/g)	6.85 ± 0.60 ^a^	2.70 ± 0.10 ^b^	2.81 ± 0.45 ^b^	<LoD
Cs (ng/g)	328.0 ± 1.8 ^a^	174.9 ± 7.7 ^b^	140.92 ± 0.34 ^c^	130.0 ± 5.9 ^c^
Li (ng/g)	20.4 ± 5.2 ^a,b^	26.9 ± 4.7 ^a^	2.09 ± 0.13 ^b^	<LoD
Ni (µg/g)	0.575 ± 0.018 ^b^	0.980 ± 0.017 ^a^	0.246 ± 0.018 ^d^	0.338 ± 0.012 ^c^
Pb (ng/g)	30.3 ± 2.8 ^a^	44 ± 11 ^a^	<LoD	<LoD
Rb (µg/g)	34.20 ± 0.29 ^a^	34.12 ± 0.43 ^a^	17.76 ± 0.28 ^c^	23.37 ± 0.48 ^b^
Sb (µg/g)	11.6 ± 2.1 ^a^	<LoD	<LoD	<LoD
Sn (ng/g)	229 ± 36 ^a^	16 ± 11 ^b^	10.9 ± 7.0 ^b^	<LoD
Sr (µg/g)	30.84 ± 0.21 ^b^	12.50 ± 0.36 ^d^	45.81 ± 0.98 ^a^	20.89 ± 0.85 ^c^
Te (µg/g)	<LoD	<LoD	<LoD	<LoD
Ti (µg/g)	6.16 ± 0.38 ^b^	8.39 ± 0.22 ^a^	6.718 ± 0.094 ^b^	6.70 ± 0.36 ^b^
V (µg/g)	<LoD	<LoD	<LoD	<LoD
W (µg/g)	<LoD	<LoD	<LoD	<LoD
Zr (ng/g)	167 ± 18 ^a^	23.0 ± 2.7 ^b^	6.2 ± 2.8 ^b^	5.8 ± 7.0 ^b^
Macro elements				
Ca (mg/g)	7.93 ± 0.77 ^a^	12.7 ± 3.0 ^a^	8.766 ± 0.062 ^a^	9.77 ± 0.37 ^a^
K (mg/g)	60.4 ± 5.2 ^a^	53.7 ± 4.0 ^a,b^	49.95 ± 7.8 ^a,b^	41.2 ± 5.2 ^b^
Mg (mg/g)	2.86 ± 0.13 ^b^	2.68 ± 0.26 ^b^	4.40 ± 0.13 ^a^	4.12 ± 0.31 ^a^
Na (µg/g)	499.8 ± 5.0 ^b^	586 ± 26 ^a^	398.4 ± 4.7 ^c^	407.7 ± 6.8 ^c^

Results expressed in µg/g (Fe, B, Al, Ti, V, Mn, Ni, Cu, Zn, Se, Rb, Sr, Mo, Te, Bi, Na), ng/g (Li, Be, Cr, Co, As, Zr, Cd, Sn, Cs, Ba, W and Pb) and mg/g (K, Ca, Mg) of dw. Values are presented as mean ± standard deviation (*n* = 3). Values that do not share the same letter are significantly different (*p* < 0.05), determined by ANOVA followed by the Tukey HSD test. AP—Aliança peels, FP—Formosa peels, AS—Aliança seeds, FS—Formosa seeds. LoD—Level of Detection. n.d.—not detected.

**Table 3:** Vitamin E profile of papaya by-products obtained from Aliança and Formosa varieties. Results are expressed in mg/100 g dw.

Vitamer	AP	FP	AS	FS
α-tocopherol	30.91 ± 1.15 ^b^	36.00 ± 0.55 ^a^	3.38 ± 0.02 ^c^	2.90 ± 0.09 ^c^
β-tocopherol	0.65 ± 0.04 ^b^	2.45 ± 0.02 ^a^	n.d.	n.d.
γ-tocopherol	24.43 ± 0.86 ^b^	39.18 ± 0.39 ^a^	0.69 ± 0.00^c^	0.64 ± 0.02 ^c^
γ-tocotrienol	6.84 ± 0.28 ^b^	11.23 ± 0.03 ^a^	n.d.	n.d.
δ-tocopherol	2.21 ± 0.12 ^b^	11.76 ± 0.17 ^a^	n.d.	n.d.
Total vitamin E	65.04 ± 1.98 ^b^	100.62 ± 0.73 ^a^	4.07 ± 0.02 ^c^	3.54 ± 0.09 ^c^

Values are presented as mean ± standard deviation (*n* = 3). Values that do not share the same letter are significantly different (*p* < 0.05), determined by ANOVA followed by the Tukey HSD test. AP—Aliança peels, FP—Formosa peels, AS—Aliança seeds, FS—Formosa seeds. n.d.—not detected.

**Table 4:** Fatty acid profiles (%) found in the peels and seeds fractions of *Papaya carica* L. (var. Formosa and Aliança).

Fatty Acids		AP	FP	AS	FS
Lauric	C12:0	1.37 ± 0.11 ^b^	2.34 ± 0.21 ^a^	n.d.	n.d.
Myristic	C14:0	4.43 ± 0.09 ^b^	5.03 ± 0.05 ^a^	0.22 ± 0.03 ^c^	0.20 ± 0.02 ^c^
Palmitic	C16:0	24.57 ± 0.20 ^b^	26.66 ± 0.20 ^a^	16.39 ± 0.10 ^c^	16.38 ± 0.07 ^c^
Palmitoleic	C16:1	5.43 ± 0.12 ^b^	7.69 ± 0.09 ^a^	0.43 ± 0.06 ^c^	0.36 ± 0.03 ^c^
Heptadecanoic	C17:0	0.80 ± 0.08 ^a^	0.53 ± 0.09 ^b^	n.d.	n.d.
Stearic	C18:0	3.64 ± 0.27 ^b^	3.11 ± 0.08 ^c^	4.52 ± 0.04 ^a^	4.72 ± 0.02 ^a^
Oleic	C18:1n9c	9.92 ± 0.42 ^c^	8.74 ± 0.25 ^d^	72.60 ± 0.20 ^b^	73.60 ± 0.05 ^a^
Linoleic ^1^	C18:2n6c ^1^	14.45 ± 0.41 ^a^	8.91 ± 0.29 ^b^	4.81 ± 0.22 ^c^	3.56 ± 0.05 ^d^
Arachidic	C20:0	1.02 ± 0.10 ^a^	1.13 ± 0.08 ^a^	0.40 ± 0.03 ^b^	0.37 ± 0.04 ^b^
α-Linolenic ^1^	C18:3n3 ^1^	28.14 ± 0.48 ^b^	30.28 ± 0.20 ^a^	0.16 ± 0.02 ^c^	0.20 ± 0.04 ^c^
cis-11-Eicosanoic	C20:1n9	n.d.	n.d.	0.28 ± 0.02 ^b^	0.34 ± 0.02 ^a^
Behenic	C22:0	1.83 ± 0.07 ^a^	1.79 ± 0.05 ^a^	0.18 ± 0.03 ^b^	0.25 ± 0.01 ^b^
Tricosanoic	C23:0	0.83 ± 0.13 ^a^	0.83 ± 0.03 ^a^	n.d.	n.d.
Lignoceric	C24:0	3.57 ± 0.03 ^a^	3.46 ± 0.25 ^a^	n.d.	n.d.
n6/n3		0.51 ± 0.02 ^c^	0.29 ± 0.01 ^c^	30.06 ± 4.00 ^a^	17.80 ± 3.56 ^b^
n9/n6		0.69 ± 0.04 ^c^	0.98 ± 0.04 ^c^	15.15 ± 0.69 ^b^	20.77 ± 0.29 ^a^
ΣSFA		42.06 ± 0.42 ^b^	44.88 ± 0.42 ^a^	21.71 ± 0.12 ^c^	21.92 ± 0.09 ^c^
ΣMUFA		15.35 ± 0.44 ^d^	16.43 ± 0.27 ^c^	73.31 ± 0.21 ^b^	74.30 ± 0.06 ^a^
ΣPUFA		42.59 ± 0.63 ^a^	39.19 ± 0.35 ^b^	4.97 ± 0.22 ^c^	3.76 ± 0.06 ^d^

Results expressed as a relative % of total fatty acids. ^1^ Essential fatty acid. Values are presented as mean ± standard deviation (*n* = 3). Values that do not share the same letter are significantly different (*p* < 0.05), determined by ANOVA followed by the Tukey HSD test. AP—Aliança peels, FP—Formosa peels, FS—Formosa seeds, AS—Aliança seeds. ΣSFA—sum of saturated fatty acids (C12:0 + C14:0 + C16:0 + C17:0 + C18:0 + C20:0 + C22:0 + C23:0 + C24:0), ΣMUFA—sum of monounsaturated fatty acids (C16:1 + C18:1n9c + C20:1n9), ΣPUFA—sum of polyunsaturated fatty acids (C18:2n6c + C18:3n3). AP—Aliança peels, FP—Formosa peels, AS—Aliança seeds, FS—Formosa seeds. n.d.—not detected.

**Table 5:** Quantification of bioactive compounds and assessment of antioxidant activity of papaya by-products from the two papaya varieties (Aliança and Formosa).

Fruit by-Product	TP (mg GAE/g)	TF (mg CE/g)	FRAP (µmol FSE/g)	DPPH^•^ (%)
AP	8.92 ± 0.37 ^a^	2.54 ± 0.08 ^a^	89.39 ± 6.41 ^a^	26.81 ± 1.7 ^b,c^
FP	7.99 ± 0.45 ^b^	2.27 ± 0.08 ^b^	74.30 ± 4.40 ^b^	26.16 ± 1.2 ^c^
AS	4.67 ± 0.15 ^c^	1.27 ± 0.05 ^c^	79.16 ± 1.79 ^b^	30.30 ± 1.6 ^a^
FS	4.31 ± 0.15 ^c^	1.11 ± 0.05 ^d^	67.62 ± 2.27 ^c^	28.46 ± 1.5 ^a,b^

Results are expressed in dry weight. Total phenolics expressed in mg GAE/g, flavonoids expressed in mg CE/g, FRAP expressed in µmol FSE/g and DPPH^•^ results expressed in % scavenging effect as mean ± standard deviation (*n* = 3). Values that do not share the same letter are significantly different (*p* < 0.05), determined by ANOVA followed by the Tukey HSD test. AP—Aliança peels, FP—Formosa peels, AS—Aliança seeds, FS—Formosa seeds.

## Data Availability

Data are contained within the article.
